# 2171 Exploring the relationships between acculturation, discrimination and function in older African immigrants: A dissertation study

**DOI:** 10.1017/cts.2018.284

**Published:** 2018-11-21

**Authors:** Manka Nkimbeng, Yvonne Commodore-Mensah, Sarah Szanton

**Affiliations:** Johns Hopkins University School of Medicine

## Abstract

OBJECTIVES/SPECIFIC AIMS: Acculturation and discrimination are associated with negative health outcomes including functional disability. The effect of these on functional disability in older African immigrants in the United States is not well understood. The purpose of this study is to describe and examine the experiences of acculturation, racial discrimination and functional disability in older African immigrants. METHODS/STUDY POPULATION: This study will use cross-sectional quantitative and qualitative mixed methods design. We plan to recruit 150 older (>55 years) participants through the African Immigrant Health Study and community-based organizations serving African immigrants. We will use the PROMIS physical function measure to assess functional disability, the Everyday Discrimination scale and the Psychological Acculturation scale will be used to measure discrimination and acculturation respectively. Higher scores indicate greater severity. RESULTS/ANTICIPATED RESULTS: We have recruited 12 participants so far. The mean age is 57 years and mean length of stay in the United States is 23 years. Mean disability score is 6.5 (range 1–38). Mean discrimination is 8.2 (range 4–15). The prevalent acculturation strategy of these participants (7) is marginalization (neither identified with the American nor African cultures). DISCUSSION/SIGNIFICANCE OF IMPACT: Preliminary results indicate pervasive discrimination and marginalization of study participants. Exploring these experiences can inform preventive strategies of coping and health behaviors that can decrease the negative effects of discrimination, acculturation and functional disability in African immigrants.

